# Enacting the curriculum: The interplay of pedagogical beliefs, motivation, and fidelity

**DOI:** 10.1371/journal.pone.0349102

**Published:** 2026-05-13

**Authors:** Özgür Tutal

**Affiliations:** Department of Child Development, Hakkari University, Hakkari, Türkiye; PNG National Research Institute, PAPUA NEW GUINEA

## Abstract

**Background:**

Curriculum fidelity – the balance between strict adherence to prescribed curricula and context-responsive adaptation – represents a critical challenge in educational implementation, particularly within centralized systems. While curriculum design has evolved toward constructivist, student-centered approaches, teachers must navigate tensions between mandated standards and diverse classroom realities. This study examines how teachers’ pedagogical beliefs (constructivist versus traditional) and teaching motivation (intrinsic versus extrinsic) interact to shape curriculum enactment decisions.

**Methods:**

A quantitative correlational design was employed with 424 in-service teachers from southeastern Türkiye. Participants completed validated scales measuring pedagogical beliefs, teaching motivation, and curriculum fidelity, which assessed both adherence and adaptation dimensions. Structural equation modeling with mediation analysis was conducted to test direct and indirect pathways among latent variables.

**Results:**

Constructivist beliefs were positively associated with curriculum adaptation and intrinsic motivation, while traditional beliefs showed positive associations with adherence and extrinsic motivation. Critically, intrinsic motivation was positively associated with adaptation and negatively associated with adherence. Conversely, extrinsic motivation was positively associated with adherence and negatively associated with adaptation. Mediation analysis indicated that both motivation types partially mediated the belief-fidelity relationships, revealing motivation as an important pathway linking beliefs and enactment preferences.

**Conclusion:**

The study illuminates the critical role of teachers’ teaching motivation in the relationship between pedagogical beliefs and curriculum implementation. By understanding how teaching motivation acts as a conduit between pedagogical beliefs and curriculum fidelity, educators and policymakers can develop targeted interventions to optimize curriculum effectiveness, fostering environments that support both teacher autonomy and curriculum integrity.

## Introduction

Contemporary educational leadership places a premium on curriculum design and implementation as a critical mechanism for enhancing student learning outcomes and achieving context-specific accountability metrics [1]. In an era characterized by rapid educational innovation and the ascendance of 21st-century competencies – such as critical thinking, problem-solving, and digital literacy – over rote knowledge [2,3], educational expectations have undergone a paradigm shift, reflected in evolving curricular frameworks. Recognizing the imperative to cultivate skills relevant to the information and communication age, nations are engaged in continuous curriculum revision, seeking to bolster educational quality and address contemporary needs [4]. Consequently, the tempo of curricular reform has accelerated in recent years, with educational systems worldwide striving to keep pace with the dynamic demands of a globalized and technologically advanced society [5]. This emphasis underscores the understanding that curricula must be dynamic and adaptive, capable of evolving to meet the changing needs of learners and the broader societal landscape.

While nations strive to align curricula with the exigencies of rapid change, curricular design alone does not guarantee educational efficacy. The operationalization of any curriculum, irrespective of its theoretical sophistication or meticulous planning, ultimately hinges upon the agency of teachers. Teachers, long recognized as influential agents within academic settings [6,7], are instrumental in translating curricular intent into pedagogical practice, serving as the bridge between theoretical frameworks and practical classroom application. Indeed, teachers serve as the linchpin for ensuring curricular effectiveness and efficiency [8], as their pedagogical decisions and instructional strategies directly impact student engagement, comprehension, and learning outcomes. This acknowledgment highlights the critical role of teacher expertise and judgment in the successful implementation of any curriculum, regardless of its inherent quality or perceived innovation.

The potential of an innovative and forward-thinking curriculum remains unrealized without commensurate teacher commitment [1], underscoring the salience of curriculum fidelity. Curriculum fidelity, defined as the degree to which teachers implement the curriculum as designed, ensures that educational objectives are achieved as intended by developers [9]. In essence, curriculum fidelity represents the convergence of the official and operational curricula [10], bridging the gap between prescribed guidelines and actual classroom practices. This construct encompasses both adherence and adaptation. Adherence, characterized by strict adherence to prescribed guidelines and instructional materials, promotes consistency and standardization, facilitating accurate program evaluation and accountability [11,12]. High curriculum fidelity can facilitate the accurate assessment of educational programs by providing a clear measure of how closely the implemented curriculum aligns with the intended design. This alignment is crucial for evaluating the effectiveness of curricula and making informed decisions about educational improvements. Adhering to the curriculum can also help educators meet accountability standards and demonstrate compliance with educational policies and mandates [13].

However, the practical application of curriculum often necessitates adaptation, driven by the heterogeneity of classroom contexts, teacher expertise, and student needs. Curriculum adaptation empowers teachers to tailor content, pedagogy, and assessment to enhance relevance and accessibility [9,14]. This dynamic approach acknowledges the inherent variability of educational settings and the need for a judicious balance between standardized implementation and context-sensitive modifications. Adaptation can involve changes to the content, teaching methods, or assessment strategies to enhance relevance and accessibility for students. This approach recognizes the dynamic nature of teaching and learning, acknowledging that effective education often requires a balance between adhering to established guidelines and making context-specific adjustments. The tension between curriculum fidelity and adaptation reflects a broader discourse on the interplay of standardization and flexibility in pedagogical practice. While proponents of strict fidelity emphasize its role in maintaining educational quality and coherence, advocates for adaptation underscore its importance in addressing diverse student needs and fostering meaningful learning experiences [15,16]. This ongoing debate highlights the complex challenges faced by educators in navigating the delicate balance between ensuring consistency and promoting individualized learning.

One of the aims of developing curricula is to introduce standardization in teaching practices and to ensure consistency across educational settings by specifying both the objectives and content and, in some cases, the instructional and assessment methods to be used. However, the implementation of these curricula in real classroom contexts is often complicated by regional disparities, varied institutional structures, and the diverse learning needs of students. Consequently, it may be neither feasible nor pedagogically sound to apply national curricula uniformly. This inherent tension between prescribed standards and practical realities has fueled an ongoing international debate on curriculum fidelity – namely, the extent to which teachers should strictly adhere to or adapt official curricula [17–19].

Across different educational systems, fidelity has been conceptualized on a continuum – from full adherence to flexible adaptation – depending on curriculum design, teacher autonomy, and systemic structures. For example, the U.S. has often favored “teacher-proof” curricula driven by product control and external accountability mechanisms [19–21], while many European models prioritize teacher autonomy and “process control,” where adaptation is seen as a mark of professional judgment rather than deviation [22,23]. Notably, the Finnish system resisted global trends of high-stakes testing and curriculum standardization by investing in teacher professionalism and trusting localized adaptations [24].

Türkiye, with its strongly centralized education system, mirrors this international tension. While national curricula are designed and mandated by the Ministry of National Education, leaving little room for local input [25,26], teachers frequently encounter classroom realities that necessitate curriculum adaptation [27–29]. Over the past two decades, Türkiye has introduced multiple curriculum reforms influenced by progressive educational philosophies and constructivist principles. These reforms aim to promote individualized learning and accommodate evolving student needs, yet they operate within a framework of high-stakes assessments and centralized accountability – factors that reinforce adherence and reduce pedagogical flexibility [30].

This structural contradiction results in a policy-practice gap in which teachers must navigate between policy-mandated adherence and classroom-driven responsiveness. The notion of curriculum adaptation remains relatively underexplored in Türkiye, despite increasing evidence that teachers do modify national programs in practice [22]. This duality has sparked critical questions: Are rigid programs truly effective if they are not implemented as designed? Can meaningful teaching occur without room for pedagogical discretion? These issues reflect a broader debate about professional autonomy versus institutional control in curriculum enactment. In light of this context, the current study does not focus to resolve the normative question of whether strict adherence or adaptation is inherently better. Instead, it focuses on how teachers position themselves within this continuum and how their pedagogical beliefs and teaching motivation relate to their curriculum enactment preferences.

### Factors influencing curriculum fidelity

The factors influencing curriculum fidelity are multifaceted, encompassing teacher-related characteristics, institutional factors, and broader systemic issues. Teacher characteristics, including their educational background, teaching experience, and personal beliefs about education, play a significant role in how they implement curricula. Teachers with higher levels of education and specialized training are more likely to understand the importance of curriculum fidelity and possess the skills to adapt curricula effectively without compromising their core objectives [31]. Institutional factors such as administrative support, availability of resources, and professional development opportunities also impact curriculum fidelity. Schools that provide robust support systems, including ongoing training and access to teaching materials, enable teachers to implement curricula more effectively. Additionally, collaborative school cultures that encourage teacher input and innovation can foster a more adaptive approach to curriculum implementation [14]. Systemic issues, including standardized testing pressures and centralized education policies, can either support or hinder curriculum fidelity. High-stakes testing environments often push teachers toward strict adherence to prescribed curricula to ensure their students perform well on assessments. Conversely, decentralized education systems that allow for greater teacher autonomy can encourage adaptation and innovation in curriculum implementation [13].

In the literature, it is seen that the studies conducted on the factors affecting curriculum fidelity related to teachers are generally the studies that try to determine the relationship between curriculum literacy [32–35], self-efficacy [36,37], pedagogical beliefs [36,38], curriculum orientations [35], curriculum design orientation preferences [39] and curriculum fidelity by using correlation or regression analysis. However, in addition to these, teachers’ teaching motivation, both intrinsic and extrinsic, can significantly affect their fidelity to the curriculum [40]. Intrinsic motivation, driven by a passion for teaching and a commitment to student success, is often associated with higher levels of curriculum fidelity. In contrast, extrinsic motivation, influenced by external rewards such as salary or job security, may not sustain long-term commitment to the curriculum [41,42]. Also, teachers’ pedagogical beliefs influence how they perceive and implement the curriculum, which in turn can affect their fidelity to it. For instance, teachers who hold constructivist beliefs could be more likely to adapt the curriculum to better meet their students’ needs. This adaptation can lead to innovative teaching practices and enhanced student learning outcomes [43,44].

### Teaching motivation

Motivation is the willingness to exert high levels of effort toward a particular goal [45]. The concept of motivation encompasses various theories and models that explain why individuals engage in specific behaviors. In educational settings, teacher motivation is crucial because it directly affects teaching quality and student outcomes. Self-determination theory is one prominent framework that distinguishes between intrinsic and extrinsic motivation [42]. Intrinsic motivation refers to engaging in teaching for the inherent satisfaction and fulfillment it brings, driven by personal interest and internal rewards. Intrinsically motivated teachers tend to derive pleasure from the process of teaching itself, finding joy in student interactions, the progression of student learning, and the intellectual stimulation teaching provides. This form of motivation is crucial as it fosters a deeper engagement with the teaching process, leading to innovative and dynamic teaching practices [46].

Extrinsic motivation, conversely, is driven by external rewards like salary, job stability, and professional acknowledgment. This type of motivation is apparent when individuals engage in an activity primarily for the desirable outcomes it produces rather than for the activity itself [47]. Extrinsically motivated teachers might be driven by the need to meet external standards, gain professional advancement, or secure job stability. While extrinsic motivation can effectively drive performance and goal achievement, it may not sustain long-term engagement or satisfaction in teaching. Both forms of teaching motivation are essential in understanding teachers’ commitment and enthusiasm, which subsequently influence their teaching practices and interactions with students [48,49].

### Pedagogical beliefs

Pedagogical beliefs significantly affect how educators approach their instructional duties. These beliefs can be broadly categorized into constructivist and traditional orientations [50,51]. Teachers with constructivist beliefs view learning as an active, student-centered process where knowledge is constructed through experience and interaction. Constructivist teachers see their role as facilitators who guide students through discovery and exploration, encouraging them to develop critical thinking and problem-solving skills. This approach is consistent with modern educational theories that highlight the significance of meaningful learning experiences, where students actively participate in constructing their understanding of concepts [52,53]. Constructivist educators tend to design learning activities that promote collaboration, inquiry, and real-world application. They believe that students learn best when they can relate new information to their existing knowledge and experiences. This belief leads to a teaching style that is flexible and responsive to individual student needs, fostering an atmosphere where students feel authorized to take ownership of their learning [54].

In contrast, teachers with traditional beliefs perceive learning as a passive absorption of information, where the teacher’s role is to transmit knowledge directly to students, who are expected to memorize and reproduce it. Traditional teaching methods often involve structured, teacher-centered activities where the focus is on delivering content efficiently and ensuring that students meet predefined academic standards. This approach is rooted in behaviorist theories of learning, which emphasize the importance of reinforcement and repetition in acquiring knowledge and skills [55]. Traditional educators might prioritize discipline, order, and uniformity in their classrooms, with a strong emphasis on rote learning and standardized testing. They often view their role as the primary source of knowledge, responsible for delivering content clearly and systematically. While this approach can be effective in certain contexts, it may not fully engage students or promote the development of higher-order thinking skills [52].

### The link between pedagogical beliefs and teaching motivation

Teaching motivation is fundamentally rooted in beliefs [56]. Whether teachers’ beliefs align with traditional or constructivist approaches plays a significant role in shaping the nature of their motivation, a relationship most robustly explained by Self-Determination Theory (SDT) [46,57]. SDT posits that motivation exists on a continuum from intrinsic to extrinsic, driven by the fulfillment of three basic psychological needs: autonomy, competence, and relatedness [58]. Practices that support autonomy, competence, and relatedness foster intrinsic motivation and internalization of values; controlling practices thwart these needs and shift motivation toward external/introjected regulation [59].

Constructivist pedagogy is often considered to be inherently more autonomy-supportive. In such environments, the teacher typically acts as a designer of learning experiences where students often have more choice. Consequently, the teacher may also experience a greater sense of professional autonomy and agency, which could serve as a source of intrinsic satisfaction [60]. This role might be less about “delivering” a preset curriculum and more about engaging in the intellectual co-construction of knowledge, which is likely to be more intrinsically motivating. In contrast, traditional practices can often be implemented in a more controlling manner, potentially emphasizing compliance and adherence. Even if chosen autonomously, these practices are frequently reinforced by external systems (e.g., high-stakes testing), which could contribute to a perceived loss of autonomy. This perceived loss may undermine intrinsic motivation [61] and could lead to a greater reliance on extrinsic motivators.

When a teacher observes students deeply engaged in constructivist activities, it may provide powerful feedback that enhances their sense of competence [62]. In such cases, success is often measured not solely by test scores but by observable student behaviors like collaboration and curiosity, which may be more personally rewarding. Conversely, in a traditional framework, competence tends to be defined more by external metrics like standardized test scores. This external locus for evaluation may foster more controlled forms of extrinsic motivation, where teachers might feel effective primarily when these external standards are met.

Finally, the collaborative nature of constructivist classrooms would appear to better fulfill the need for relatedness, potentially making teaching more socially rewarding. While traditional settings can also foster relatedness, it may be based more on hierarchical respect than collaborative community and might not satisfy the need for connection as thoroughly.

### The present study

The interplay of these factors highlights the complexity of achieving curriculum fidelity in diverse educational contexts. Understanding how these variables interact is crucial for designing effective educational interventions and policies that support both adherence to curricular standards and necessary adaptations to meet student needs. A structural equation model can be used to analyze the influences of these variables simultaneously and it can provide novel insights into the structural relationships. From this point of view, the current research aims to examine the relationship between teachers’ pedagogical beliefs and their teaching motivation, which is thought to be related to curriculum fidelity (the extent to which they adhere or adapt rather than their fidelity levels), based on structural equation modeling, which includes mediating variables and will provide more comprehensive and robust results than correlation or regression analysis.

**Fig 1:**
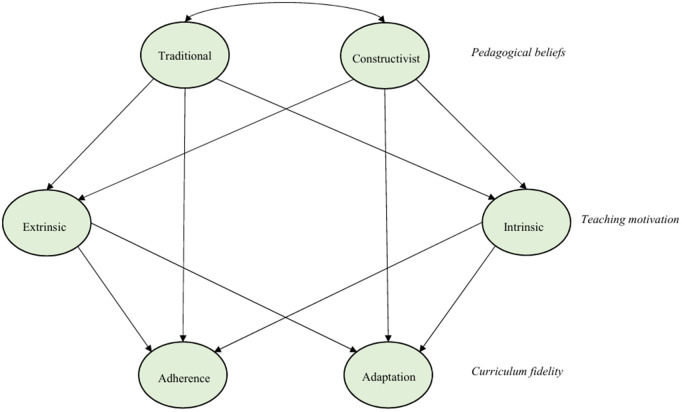
The hypothesized model.

Based on the related literature suggesting that pedagogical beliefs and teaching motivation potentially impact curriculum fidelity, it is hypothesized that teachers’ beliefs about teaching and learning, as well as their teaching motivation, can influence their fidelity to the curriculum. The hypothesized model is illustrated in Fig 1. Consequently, this study addresses the following research questions:

- RQ_1_: Are there any significant interrelationships between teachers’ pedagogical beliefs, teaching motivation, and curriculum fidelity?
- RQ_2_: Does teaching motivation mediate the relationship between pedagogical beliefs and curriculum fidelity?

## Methodology

### Ethics statement

Ethics approval was obtained from Hakkari University Scientific Research and Publication Ethics Board, which granted ethical clearance with the reference number 2024/101 on July 1, 2024.

The information sheet and informed consent were presented to the participants, informing them of the purpose of the research, confidentiality, anonymity, potential risks and their rights to withdraw at any time without prejudice. Written informed consent was obtained from all participants involved in the study. All research was performed in accordance with relevant guidelines and regulations.

### Research design

To explore the relationship among teachers’ pedagogical beliefs, teaching motivation, and curriculum fidelity a quantitative correlational design was employed. The current study hypothesized a model (Fig 1), and tested the hypothesized model through the structural equation modeling (SEM). SEM is a powerful statistical technique that allows researchers to test theoretical models that specify the relationships between observed and latent variables in a simultaneous way [63,64]. It examines the measurement properties of a variable and the interrelationships between multiple variables, often regarded as a blend of regression and factor analysis. Typically, SEM adopts a confirmatory approach, where the researcher proposes a model of relationships between variables of interest and assesses whether the observed data supports the hypothesized directionality and significance of these relationships [65]. In the context of this study, through SEM, an attempt was made to elucidate how constructivist and traditional educational beliefs along with, intrinsic and extrinsic motivations, shape teachers’ adherence to or adaptation to the prescribed curriculum. The model includes pathways representing theoretically specified directional associations, allowing for the examination of both direct and indirect relationships.

### Participants

A total of 424 in-service teachers working in the Hakkari province in southeastern Türkiye were randomly selected to participate in this study. To select the participating schools, stratified random sampling was employed, aiming for representation across all educational levels from the city center and its four districts. Questionnaires were distributed to 526 teachers across seven preschools, eight primary schools, nine middle schools, and eight high schools. A total of 424 teachers responded, yielding an 81% response rate. Teachers who volunteered to participate in the study were informed that their information would be kept confidential and used exclusively for academic purposes. Among the participants, there were 200 females (47%) and 224 males (53%). There were 32 preschool teachers (8%), 141 primary school teachers (33%), 92 middle school teachers (22%), and 159 high school teachers (37%). Table 1 displays the demographic information of the participants in more detail. Results from comparative analyses indicated no significant differences in teachers’ curriculum fidelity, teaching motivations, or pedagogical beliefs based on several demographic and professional variables. Specifically, mean scores for these constructs did not significantly vary by age (F = 0.307,p > .01; F = 0.851, p > .01; F = 1.159, p > .01, respectively), experience (F = 0.071, p > .01; F = 0.929, p > .01; F = 1.030, p > .01, respectively), work context (F = 0.410, p > .01; F = 0.381, p > .01; F = 1.698, p > .01, respectively), gender (t= −0.710, p > .01; t = 1.013, p > .01; t = 0.525, p > .01, respectively), or major (F = 0.898, p > .01; F = 1.638, p > .01; F = 1.365, p > .01, respectively) as reported in Table in S1 Table.

**Table 1:** The demographic information of the teachers.

Variable	Frequency	Percentage	Variable	Frequency	Percentage
*Age*			*Gender*		
20-24	15	3.5	Female	200	47.2
25-29	125	29.5	Male	224	52.8
30-34	105	24.8	*Major*		
35-39	117	27.6	Primary school teacher	103	24.3
40+	62	14.6	Social sciences	66	15.6
*Experience*			Preschool teacher	38	9.0
0-4 years	153	36.1	Mathematics	36	8.5
5-9 years	114	26.9	Turkish	32	7.5
10-14 years	74	17.5	Vocational courses	32	7.5
15-19 years	51	12.0	Science	30	7.1
20 + years	32	7.5	Special education	30	7.1
*Work context*			Foreign language	27	6.4
Preschool	32	7.5	Physical education	11	2.6
Primary school	141	33.2	Arts	10	2.3
Middle school	92	21.7	Information technologies	9	2.1
High school	159	37.5			

### Measures

The main instrument used to collect data for this study was a structured survey. This was conducted over four months, from July 4 to November 12, 2024. The survey used to collect the study data consisted of four sections: (1) teacher profile, (2) teaching motivation, (3) pedagogical beliefs, and (4) curriculum fidelity.

Motivation to Teach Scale (MTS), was developed by Kauffman et al. [66] and adapted into Turkish by Ayık et al. [67]. The scale was used to measure teachers’ motivation to teach in terms of intrinsic motivation (seven items, α = .86, e.g., “I teach because I believe it will give me a sense of deep personal fulfillment.”), and extrinsic motivation (five items, α = .76, e.g., “I chose teaching because benefits are good.”). This instrument used a 6-point Likert scale, with 1 denoting “strongly disagree” and 6 denoting “strongly agree.”

Teaching and Learning Conceptions Scale (TLCS), developed by Chan and Elliot [52]. In this study, instead of the original form of the scale consisting of 30 items, the short form adapted to Turkish by Doruk [68] was employed. The short form of the scale has 14 items on a 5-point response scale, ranging from 1 (strongly disagree) to 5 (strongly agree), with two components: constructivist conception (seven items, α = .85, e.g., “Good teachers always encourage students to think for answers themselves.”), and traditional conception (seven items, α = .81, e.g., “The major role of a teacher is to transmit knowledge to students.”).

Curriculum Fidelity Scale (CFS), developed by Öner-Sunkur and Yılmaz [33] to measure teachers’ level of curriculum fidelity in two dimensions: adherence (eleven items, α = .89, e.g., “I am willing to implement the curriculum in its original form.”), and adaptation (eleven items, α = .89, e.g., “I adapt the curriculum to the traditions and customs of the students.”). The teachers who participated in the study rated their level of agreement using a 5-point Likert scale, with 5 indicating ‘strongly agree’ and 1 indicating ‘strongly disagree.’

### Data analysis

Preliminary monitoring of the collected data revealed no missing entries. To verify the factor structure of the observed variables, confirmatory factor analyses (CFAs) were conducted on the three instruments using the Analysis of Moment Structure (AMOS) version 22 with the maximum likelihood estimation approach. Afterward, structural equation modeling (SEM) was conducted to examine the structural relationships between teachers’ pedagogical beliefs and curriculum fidelity, and their subcomponents. Mediation analysis evaluated the impacts of two subcomponents of teaching motivation in the relationship between pedagogical beliefs and curriculum fidelity. The analysis employed an advised 5000 bootstrap sample to specify the significance level for loadings, weights, and path coefficients [69].

## Results

### Descriptive statistics

Primarily, descriptive statistics were checked. As indicated in Table 2, the teachers’ mean scores for the sub-dimensions of the scales ranged between 2.442 and 4.384. The standard deviations ranged between 0.569 and 1.283, reflecting narrow spreads around the mean scores. The Cronbach alpha (α) coefficients estimated for the intrinsic motivation, adherence, adaptation, constructivist and traditional beliefs were at ideal levels, and for the extrinsic motivation, it was at acceptable level, indicating that internal consistency was sufficient.

**Table 2:** Descriptive statistics.

	Mean	SD	Range	Low	High	α
Extrinsic	3.375	1.283	5.00	2	12	.673
Intrinsic	4.318	1.101	5.00	6	36	.851
Constructivist	4.384	.569	4.00	7	35	.879
Traditional	2.442	.870	4.00	5	25	.838
Adherence	3.066	.826	4.00	6	30	.855
Adaptation	3.975	.616	3.91	12	55	.915

As seen in Table 3, the correlation analysis revealed that the traditional and constructivist sub-dimensions of TLCS demonstrated a significant negative association (r = −0.264, p < .01), reflecting their divergent pedagogical orientations. Traditional beliefs showed weak but significant links with adherence (r = 0.230, p < .01) and extrinsic motivation (r = 0.159, p < .01), suggesting a preference for structured, reward-driven instruction. In contrast, constructivist beliefs aligned positively with adaptation (r = 0.386, p < .01) and intrinsic motivation (r = 0.363, p < .01), highlighting their affinity for flexible, student-centered practices. The MTS further illuminated this divide: Intrinsic motivation exhibited robust ties to adaptation (r = 0.408, p < .01) and constructivist beliefs (r = 0.363, p < .01), while extrinsic motivation correlated significantly with adherence (r = 0.316, p < .01) weakly with traditional beliefs (r = 0.159, p < .01). CFS sub-dimensions diverged sharply – adherence and adaptation were unrelated (r = −0.095, p > .01). Adherence linked to traditional beliefs (r = 0.230, p < .01) and extrinsic motivation (r = 0.316, p < .01), whereas adaptation showed a striking association with constructivist beliefs (r = 0.386, p < .01) and intrinsic motivation (r = 0.408, p < .01). Together, these findings paint a coherent portrait: Traditional beliefs-Extrinsic motivation-Adherence and Constructivist beliefs-Intrinsic motivation-Adaptation emerge as parallel pathways, each shaping educators’ practices through distinct philosophical and motivational lenses.

**Table 3:** Correlation coefficients among constructs.

	Traditional	Constructivist	Adherence	Adaptation	Intrinsic	Extrinsic
**Traditional**	1	−.264^*^	.230^*^	−.081	.033	.159^*^
**Constructivist**	−.264^*^	1	.016	.386^*^	.363^*^	.071
**Adherence**	.230^*^	.016	1	−.095	−.029	.316^*^
**Adaptation**	−.081	.386^*^	−.095	1	.408^*^	−.044
**Intrinsic**	.033	.363^*^	−.029	.408^*^	1	.389^*^
**Extrinsic**	.159^*^	.071	.316*	−.044	.389^*^	1

* p < .01

The participation of teachers from diverse schools introduces a potential risk of biased results due to data clustering [70]. To examine this, Intra-class Correlation Coefficients (ICCs) were computed for all outcome variables by dividing the between-group variance by the sum of the between-group and within-group variance [71,72]. This determines whether the data exhibited a clustered structure. As presented in the Table 4, all ICC values were below the 0.25 threshold (Traditional beliefs = 0.18; Constructivist beliefs = 0.05; Intrinsic motivation = 0.19; Extrinsic motivation = 0.11; Adherence = 0.25; Adaptation = 0.25).

**Table 4:** Intra-class correlation coefficients by schools.

Variable	Between-groupvariance	Within-groupvariance	ICC
Traditional	56.3	263.8	.176
Constructivist	7.4	129.7	.054
Adherence	71.7	217.5	.248
Adaptation	39.9	120.6	.249
Intrinsic	96.4	416.1	.188
Extrinsic	73.4	623.0	.105

Furthermore, because the teachers were drawn from different educational levels (e.g., preschool, primary), ICCs were also estimated across school levels (Table 5). These analyses similarly revealed that all ICCs were below 0.25 (Traditional beliefs = 0.05; Constructivist beliefs = 0.01; Intrinsic motivation = 0.01; Extrinsic motivation = 0.03; Adherence = 0.03; Adaptation = 0.04). Collectively, these results indicate that the observations were independent [73]. Consequently, the use of multilevel structural equation modeling was not warranted for this dataset [74,75].

**Table 5:** Intra-class correlation coefficients by educational levels.

Variable	Between-groupvariance	Within-groupvariance	ICC
Traditional	17.7	320.1	.052
Constructivist	1.9	137.1	.014
Adherence	10.1	289.2	.034
Adaptation	5.9	160.5	.036
Intrinsic	4.8	512.5	.009
Extrinsic	19.7	696.4	.027

### Confirmatory factor analysis of the MTS, TLCS, and CFS

Confirmatory factor analyses (CFAs) of the three instruments were conducted prior to SEM path analysis. Items with factor loadings above 0.58, the established minimum threshold, were included in the model. Model fit was evaluated using the root mean squared error of approximation (RMSEA), standardized root mean square residual (SRMR), comparative fit index (CFI), goodness of fit index (GFI), and Tucker-Lewis index (TLI). The acceptable range for χ2/df is between 2 and 5, and for RMSEA and SRMR, it is between 0.05 and 0.08. Additionally, TLI, CFI, and GFI values are considered acceptable within the range of 0.90 to 0.95 [63,76–78]. As seen in Table 6, the CFA model fits for MTS, TLCS, and CFS were acceptable.

**Table 6:** Model fit indices for CFA models.

	X^2^/df	GFI	CFI	TLI	SRMR	RMSEA	p
MTS	3.80	.97	.96	.94	.04	.08	<.001
TLCS	2.25	.96	.97	.96	.05	.05	<.001
CFS	3.73	.90	.92	.90	.06	.08	<.001

The CFA results also revealed that the indicators’ correlations with their proposed latent components ranged from 0.34 to 0.72, indicating medium to large effect sizes [79]. The standardized coefficients (β) ranged from 0.58 to 0.85, all of which were statistically significant at p < .001. The critical ratios (C.R.) varied between 7.39 and 15.90. Furthermore, both the composite reliability (CR) and average variance extracted (AVE) were used to assess internal consistency reliability and convergent validity. According to Hair et al. [69], a CR value of 0.60 and an AVE value of 0.50 or above are considered adequate. Additionally, a standardized estimate greater than 0.50 sufficiently explains its latent variable. The results indicated that the CR, AVE, and standardized estimates met the required criteria (Table 7).

**Table 7:** Results generated from CFA.

	Domains	R^2^	β	C.R.	p	AVE	CR
MTS	Extrinsic	.48−.53	.69−.73	7.39-7.39	<.001	.52	.68
	Intrinsic	.36−.58	.60−.76	10.98-14.85	<.001	.50	.85
TLCS	Constructivist	.43−.59	.66−.77	12.00-13.84	<.001	.52	.88
	Traditional	.35−.72	.59−.85	10.22-13.29	<.001	.52	.84
CFS	Adherence	.38−.66	.61−.81	11.99-15.90	<.001	.50	.86
	Adaptation	.34−.58	.58−.76	11.49-13.06	<.001	.50	.92

### Common method bias assessment

Given that all data were collected via single-wave, self-report surveys, a Harman’s single-factor test was conducted to assess the potential influence of common method bias [80]. An unrotated exploratory factor analysis was performed on all items from the three scales. The first unrotated factor accounted for 22.98% of the total variance, well below the 50% threshold [81]. This suggests that common method bias is unlikely to be a substantial threat to the validity of the observed relationships.

### Findings from SEM analysis

**Fig 2:**
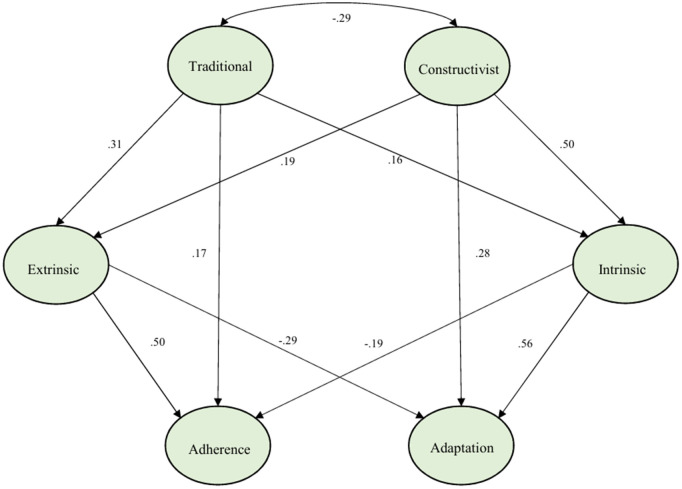
Structural equation modeling of the relations between teachers’ pedagogical beliefs, teaching motivation, and curriculum fidelity.

To examine the path correlations among pedagogical beliefs, teaching motivation, and curriculum fidelity, path analysis was conducted using SEM. The hypothesized mediation model is grounded in self-determination theory [42] and prior research linking beliefs to motivation and behavior [56]. Teachers’ pedagogical beliefs shape their perceptions of teaching roles, which in turn influence their motivational orientations [82–84]. Motivation then affects behavioral outcomes, such as curriculum implementation [85–88]. While acknowledging the cross-sectional nature of the data, the theoretical ordering – from beliefs → motivation → fidelity – is supported by empirical studies in teacher education and the results of the correlation analysis conducted within the scope of the current study, presented in Table 3 (e.g., low and insignificant relationships among traditional beliefs – adaptation, constructivist beliefs – adherence, traditional beliefs – intrinsic motivation and constructivist beliefs – extrinsic motivation). In addition, the lack of significant differences between the groups as a result of the comparison tests (Table in S1 Table) showed that a single-level SEM is also acceptable. The SEM results, shown in Fig 2, revealed the structural relationships between the latent variables.

The fit indices for the structural model were as follows: χ2/df = 2.45, CFI = 0.89, TLI = 0.88, SRMR = 0.08, and RMSEA = 0.06. While the CFI and TLI values fall slightly below the conventional 0.90 threshold recommended by Hu and Bentler [77], several considerations support the adequacy of model fit given the complexity of the present model. First, the SRMR meets the recommended cutoff, and the RMSEA falls within the acceptable range [63,77]. Second, research has shown that CFI and TLI tend to be suppressed in more complex models (e.g., those with 6 latent variables and 37 indicators) relative to the simpler models used to derive conventional cutoffs [89,90]. Third, with a sample size of 424 and a model of this complexity, the combination of acceptable RMSEA, SRMR, and χ2/df collectively indicates that the model provides a reasonable approximation to the data [64,69]. Therefore, the model was retained for interpretation of path coefficients.

The results of the path analysis are presented in Table 8. The constructivist belief had significant positive relations with intrinsic motivation, extrinsic motivation, and adaptation (β = 0.504, 0.190, and 0.277 respectively, p < 0.01). The traditional belief also had significant positive relations with intrinsic motivation, extrinsic motivation, and adherence (β = 0.156, 0.305, and 0.173 respectively, p < 0.01). In addition, while intrinsic motivation had positive significant relations with adaptation (β = 0.564, p < 0.01), it had negative significant relations with adherence (β = −0.188, p < 0.01). Besides that, while extrinsic motivation had positive significant relations with adherence (β = 0.497, p < 0.01), it had negative significant relations with adaptation (β = −0.290, p < 0.01).

**Table 8:** Results of the path analysis.

Path	B	β	SE	p
Constructivist → Intrinsic	1.025	.504	.131	.000
Traditional → Extrinsic	.419	.305	.096	.000
Constructivist → Extrinsic	.342	.190	.118	.004
Traditional → Intrinsic	.242	.156	.086	.005
Intrinsic → Adaptation	.247	.564	.028	.000
Extrinsic → Adherence	.418	.497	.060	.000
Extrinsic → Adaptation	−.143	−.290	.029	.000
Intrinsic → Adherence	−.140	−.188	.040	.000
Constructivist → Adaptation	.248	.277	.051	.000
Traditional → Adherence	.200	.173	.063	.001

### Testing of mediation

As for the mediation effects, there were four specific indirect path coefficients in the model. The results showed that all four indirect path coefficients were significant; two were significantly positive, whereas another two were significantly negative (Table 9). Extrinsic motivation was significantly associated with the relationship between traditional belief and adherence (β = 0.130, t = 3.084, p < .01). Intrinsic motivation was significantly associated with the relationship between constructivist belief and adaptation (β = 0.183, t = 4.485, p < .01). On the other hand, intrinsic motivation was significantly associated with the relationship between traditional belief and adherence (β = −0.030, t = 3.084, p = .01). Besides, extrinsic motivation was significantly associated with the relationship between constructivist belief and adaptation (β = −0.054, t = 4.485, p < .01).

**Table 9:** Testing of mediation.

Relationships	Direct Effect^*^	Indirect Effect^*^	Bias-corrected CI %95^*^		p	Conclusion
**Lower**	**Upper**
Traditional→Extrinsic→Adherence	.194 (3.084)	.130	.051	.243	.001	Partial statistical mediation
Traditional→Intrinsic→Adherence	.194 (3.084)	−.030	−.081	−.006	.010	Partial statistical mediation
Constructivist→Intrinsic→Adaptation	.244 (4.485)	.183	.124	.272	.000	Partial statistical mediation
Constructivist→Extrinsic→Adaptation	.244 (4.485)	−.054	−.107	−.022	.001	Partial statistical mediation

* Unstandardized coefficients reported. Values in parentheses are t- values.

## Discussion

This study utilized SEM to investigate the relationship among pedagogical beliefs, teaching motivation, and curriculum fidelity. The results indicate that teachers’ pedagogical beliefs are statistically associated with their curriculum fidelity, consistent with the hypothesized directional model. According to Zhu et al. [91], factors such as teachers’ adopted educational philosophy, teaching skills, and motivation are key issues within the concept of curriculum fidelity, and teachers’ values, beliefs, and personal preferences determine how the curriculum is implemented in school contexts. Similarly, Anderson [92] asserted that teachers’ beliefs are closely linked to curriculum implementation. According to the results shown in Table 6, constructivist beliefs are positively related to adaptation, whereas traditional beliefs are positively correlated with adherence. The findings by Baş and Şentürk [38] indicate that teachers with constructivist teaching-learning conceptions exhibit higher levels of curriculum fidelity. Similarly, Yılmaz and Kahramanoğlu [35] found that a constructivist curriculum orientation is a positive and significant predictor of teachers’ curriculum fidelity. Rovegno and Bandhauer [93] reported that teachers who value and believe in a constructivist approach can successfully adapt the curriculum even in disadvantaged contexts. Shawer et al. [94] claimed that teachers using the adaptation approach create more engaging educational activities compared to those employing the adherence approach, which is consistent with constructivist principles. In contrast to adherence perspectives, adaptation approaches view teachers as learners and active members of the classroom community, as envisaged by constructivism [95]. This perspective allows teachers to adapt to changing conditions [96]. Çeliker-Ercan and Çubukçu [97] suggested that the philosophy of the curricula developed based on constructivism encourage teachers to adapt curriculum materials despite pressures to adhere strictly. Conversely, traditional beliefs limit teachers’ roles to transmitters or receivers of the curriculum [98], treating teachers as communication tools [99], and reducing their roles in the classroom [100], which aligns with an adherence approach based on the positivist paradigm.

When the relationship between pedagogical beliefs and teaching motivation is examined, the results of the study revealed that both constructivist and traditional beliefs have a positive and significant relationship with both intrinsic and extrinsic motivation. The current research supports Baş’s [82] finding, indicating that teaching and learning beliefs positively and significantly predict teaching motivation. Teaching motivation has belief-related roots [56]. According to Richardson [101], beliefs are a subset of constructs that drive individual actions. In this context, pedagogical beliefs influence teachers’ classroom behaviors and practices [102] as well as their teaching motivation [84]. Although constructivist beliefs impact both intrinsic and extrinsic motivation, this study found a stronger relationship with intrinsic motivation. Conversely, traditional beliefs were more strongly linked to extrinsic motivation. Roth et al. [103] indicated that teachers’ motivation is associated with student-centered or productive teaching styles. Similarly, research by Baş and Baştuğ [83] reported a positive and significant correlation between constructivist beliefs and intrinsic motivation, while traditional beliefs had a higher correlation with extrinsic motivation compared to intrinsic motivation. The results of Yıldızlı et al. [104] also reveal significant positive correlations between traditional beliefs and extrinsic motivation and between constructivist beliefs and intrinsic motivation. Furthermore, an examination of their model’s path coefficients indicates that traditional beliefs more strongly predict extrinsic motivation, whereas constructivist beliefs are a stronger predictor of intrinsic motivation, which aligns with the findings of the present study. Moreover, Huang et al. [105] found that pre-service teachers with stronger constructivist teaching beliefs also reported greater intrinsic motivation for a future teaching career.

Another relationship examined in this research was between teachers’ teaching motivation and curriculum fidelity. Intrinsic motivation showed a positive association with adaptation and a negative association with adherence. This pattern, while cross-sectional, is consistent with the interpretation that teachers with higher intrinsic motivation tend to report greater adaptation, though causal claims require longitudinal evidence. Consequently, the observed negative relationship between intrinsic motivation and adherence is conceptually justified. Conversely, extrinsic motivation had a positive significant relationship with adherence and a negative significant relationship with adaptation. This suggests that when teachers are driven primarily by extrinsic sources, they tend to adhere strictly to the curriculum rather than adapt it. The negative relationship is substantively appropriate, as high extrinsic motivation likely diminishes teachers’ inclination toward adaptation. These interpretations are further supported by the correlation coefficients presented in Table 3. Aytaç [85] also concluded that both intrinsic and extrinsic motivation are positively and significantly correlated with curriculum fidelity. Teaching motivation impacts classroom teaching practices during the educational process [88]. Ransford et al. [87] discovered that psychological factors such as motivation influence curriculum implementation. Furthermore, Bond et al. [86] asserted a relationship between motivation and curriculum fidelity, and Bay et al. [39] highlighted that teachers’ motivation to teach plays a crucial role in curriculum fidelity.

To further explore the relationships among the variables in the hypothesized model, mediation analysis was conducted. The results of the mediation analysis showed that both intrinsic and extrinsic motivation partially mediated the relationships between constructivist belief and adaptation, and traditional belief and adherence, as all direct and indirect effects were statistically significant [65,106]. Specifically, extrinsic motivation acted as a complementary mediator for the direct effect between traditional belief and adherence, as both the direct and indirect effects pointed in the same direction. However, for the relationship between constructivist belief and adaptation, extrinsic motivation served as a competitive mediator because the direct and indirect effects pointed in opposite directions [107]. On the other hand, intrinsic motivation was a complementary mediator for the relationship between constructivist belief and adaptation, while it was a competitive mediator for the relationship between traditional belief and adherence.

One finding that warrants specific theoretical attention is the positive, albeit modest, direct path from traditional pedagogical beliefs to intrinsic motivation (β = 0.156, p = .005). At first glance, this appears to contradict self-determination theory’s premise that controlling, teacher-centered practices thwart the basic psychological needs for autonomy and competence, thereby undermining intrinsic motivation [59]. However, several considerations reconcile this finding with SDT. First, the magnitude of this effect (β = 0.156) is substantially smaller than that of constructivist beliefs on intrinsic motivation (β = 0.504), indicating that traditional beliefs are far less supportive of intrinsic motivation. SDT does not predict that controlling contexts eliminate intrinsic motivation entirely, but rather that they undermine it relative to autonomy-supportive contexts [108]. The pattern of coefficients is therefore consistent with SDT expectations. Second, SDT emphasizes that need satisfaction depends on the individual’s subjective experience of autonomy, competence, and relatedness, not on the objective structure of the activity [109]. In the highly centralized Turkish educational context, where national curricula are mandated by the Ministry of National Education and high-stakes assessments shape instructional priorities [22,25], teachers who master the prescribed curriculum and implement it skillfully may experience genuine competence satisfaction. When this mastery is autonomously chosen rather than merely compliant, it can contribute to intrinsic motivation. As Roth et al. demonstrated, teacher motivation varies along the autonomy continuum regardless of the specific teaching style employed [103]. Third, the traditional and constructivist belief subscales showed only a modest negative correlation (r = −0.264), suggesting that teachers in this sample do not hold mutually exclusive belief systems. Rather, many teachers maintain “eclectic” belief profiles that incorporate elements of both orientations [110]. The positive path from traditional beliefs to intrinsic motivation may reflect this eclecticism: teachers who endorse traditional beliefs may simultaneously value certain student-centered practices, and it is this blended orientation that supports intrinsic motivation. The CFA results supported the discriminant validity of the two belief subscales (AVEtrad = .52, AVEconst = .52; squared inter-construct correlation = .070, below the AVE of each construct), indicating that the modest correlation reflects conceptual overlap rather than measurement failure. Finally, the TLCS traditional subscale measures epistemological beliefs about the nature of learning (e.g., “Learning primarily involves memorizing facts”) rather than interpersonal controlling behaviors [52]. Holding traditional epistemological beliefs does not necessarily entail a controlling teaching style. A teacher who believes that knowledge transmission is important can still deliver instruction in an autonomy-supportive manner, providing choice, rationale, and acknowledgment of student perspectives [108]. Thus, traditional beliefs may not directly translate into need-thwarting practices.

### Limitations and future recommendations

This study has several limitations that should be acknowledged. First, the data were gathered through self-reported surveys, similar to previous research. While this approach allows for a larger sample size, it may also lead to discrepancies between teachers’ perceptions. Future research should incorporate additional methods such as interviews and observations to triangulate the data, thereby enhancing our understanding of the relationships between teachers’ pedagogical beliefs, teaching motivation, and curriculum fidelity.

Second, a cross-sectional design may limit the study’s capacity to capture the dynamic nature of curriculum fidelity over time, whereas a longitudinal approach would likely yield more comprehensive understanding. Consequently, while the SEM results are consistent with the hypothesized causal ordering (beliefs → motivation → fidelity), causal claims are avoided, and all path coefficients are instead interpreted as directional associations for which testing with longitudinal or experimental designs is required.

Third, the sample was drawn exclusively from Hakkari province in southeastern Türkiye, which has distinctive socioeconomic, demographic, and geopolitical characteristics that substantially constrains generalizability at multiple levels. Consequently, the findings may not be representative of teachers in other regions of Türkiye (e.g., western or central Anatolia, metropolitan areas, or provinces with different socioeconomic and security profiles). Furthermore, the unique cultural, geopolitical, and educational characteristics of Hakkari limit generalization to other national or cultural contexts entirely. Future research should address these limitations by: (a) employing stratified sampling across multiple provinces and geographic regions within Türkiye to test regional generalizability; (b) conducting cross-cultural replication studies in different national contexts (e.g., centralized vs. decentralized systems, collectivist vs. individualist cultures).

Fourth, this study did not include demographic factors like gender, teaching experience, work environment, and academic major in the analysis. Although this study found no significant differences between groups in demographic variables and employed a single-level model, future research using larger and more diverse samples should utilize multilevel structural equation modeling to examine cross-level associations. The observed positive correlation between intrinsic and extrinsic motivation (r = .389, p < .01) also warrants comment. While self-determination theory positions these as distinct motivational orientations that can co-exist [42], common method bias could potentially inflate this association. However, the Harman’s single-factor test indicated that common method bias is unlikely to substantively bias the findings. Moreover, the discriminant validity of the two motivation subscales was supported by the CFA results, with the AVE for each construct exceeding the squared inter-construct correlation (AVEintrinsic = .50, AVEextrinsic = .52; r² = .151), satisfying the Fornell-Larcker criterion. Lastly, it is recommended that future studies include more contextual variables, such as attitudes and self-efficacy, to provide a deeper insight into these dynamics.

### Implications

#### Theoretical implications.

The findings underscore the significant interplay between teachers’ pedagogical beliefs and their teaching motivation. Constructivist and traditional beliefs both positively influence intrinsic and extrinsic motivation, though constructivist beliefs have a stronger link to intrinsic motivation. This aligns with the theoretical perspective that educational beliefs shape motivational orientations [56]. The study enriches the understanding that teaching philosophies not only dictate pedagogical approaches but also impact the motivational drives behind teaching practices.

The positive correlations between constructivist beliefs and adaptation, and traditional beliefs with adherence, reinforce existing theories on curriculum fidelity [93,94]. This study extends the theoretical framework by demonstrating how motivational factors mediate these relationships. The dual role of intrinsic and extrinsic motivation as both direct influencers and mediators of curriculum fidelity processes provides a nuanced understanding of how teachers’ internal states affect their fidelity to curriculum models.

The mediation analysis highlights intrinsic and extrinsic motivation as partial mediators in the relationship between pedagogical beliefs and curriculum fidelity. This theoretical implication suggests that while beliefs directly influence fidelity, the motivational orientation can enhance or mitigate this influence, which adds a layer of complexity to existing educational theories on curriculum implementation.

#### Practical implications.

The results suggest that teacher training programs should emphasize the development of both constructivist and traditional teaching-learning conceptions to foster balanced motivational profiles. Training that enhances intrinsic motivation can encourage adaptive approaches to curriculum, while programs focusing on extrinsic motivation might support adherence to prescribed curricula. Tailoring professional development to align with teachers’ motivational orientations can enhance curriculum fidelity and overall teaching effectiveness.

Curriculum designers should consider the motivational impacts of curriculum structures. Curricula that support constructivist approaches might benefit from strategies that enhance intrinsic motivation, thereby promoting adaptive teaching practices. Conversely, curricula requiring strict adherence might be more effective if they incorporate elements that bolster extrinsic motivation. Understanding the motivational drivers behind teachers’ curriculum fidelity can lead to more effective and sustainable educational practices.

Educational policies should recognize the dual role of pedagogical beliefs and motivation in curriculum fidelity. Policies that provide flexibility and support for intrinsic motivational factors may foster better adaptation and implementation of curricula. Additionally, policies that incentivize extrinsic motivation can help maintain adherence where necessary. By integrating motivational considerations into policy frameworks, educational systems can achieve a more effective alignment between teaching practices and curriculum goals.

#### Implications of borderline model fit.

The structural model yielded CFI = 0.89 and TLI = 0.88, values slightly below the conventional 0.90 threshold. While this indicates that the model does not perfectly reproduce the observed covariance structure, several considerations support confidence in the reported path coefficients and mediation estimates.

First, simulation research suggests that modest misspecification in global fit indices does not necessarily bias individual parameter estimates, particularly when the misspecification is diffuse rather than concentrated in specific paths [111]. In the present model, the pattern of fit – acceptable RMSEA and SRMR alongside marginally low CFI/TLI – suggests that the discrepancy between model and data is distributed across many small residuals rather than reflecting a major structural omission.

Second, the theoretical coherence of the findings strengthens confidence in their validity. The results reveal a clear double-dissociation pattern: constructivist beliefs → intrinsic motivation → adaptation, and traditional beliefs → extrinsic motivation → adherence. This pattern is precisely what self-determination theory would predict and is unlikely to emerge as an artifact of borderline model fit. If model misspecification were substantively biasing the results, one would expect a less theoretically meaningful pattern of coefficients.

Third, the bootstrap confidence intervals for the indirect effects (Table 9) provide robust evidence for the mediation pathways. All bias-corrected confidence intervals excluded zero, and the significance levels (p < .01 for all indirect effects) indicate that the mediation findings are reliable even accounting for sampling variability. Bootstrap methods are less sensitive to distributional assumptions and modest model misspecification than traditional standard errors [69,112].

Finally, the cross-sectional design precludes causal claims regardless of fit indices, as noted in the limitations. The borderline fit does not undermine the primary contribution of this study: demonstrating that motivation serves as a statistical mediator between beliefs and fidelity in a theoretically specified direction, warranting longitudinal investigation.

## Conclusion

This study provides valuable insights into the dynamic relationships between pedagogical beliefs, teaching motivation, and curriculum fidelity. The findings highlight the significant role of both intrinsic and extrinsic motivation in mediating the associations between pedagogical beliefs and curriculum fidelity within the hypothesized model. By elucidating these complex interactions, the study contributes to both theoretical understandings and practical applications in the field of education. The implications for teacher training, curriculum design, and educational policy underscore the importance of considering motivational factors in educational practices. Future research could explore longitudinal impacts and extend these findings across different educational contexts to further validate and expand upon the current study’s conclusions.

## Supporting information

S1 Table Results generated from comparative analyses of teachers’ curriculum fidelity, teaching motivations, and pedagogical beliefs based on demographic variables. (DOCX)

## Abbreviations

SDT: Self-Determination Theory
SEM: Structural Equation Modeling
MTS: Motivation to Teach Scale
TLCS: Teaching and Learning Conceptions Scale
CFS: Curriculum Fidelity Scale
CFA: Confirmatory Factor Analysis
ICC: Intra-class Correlation Coefficient
RMSEA: Root Mean Squared Error Of Approximation
SRMR: Standardized Root Mean Square Residual
CFI: Comparative Fit Index
GFI: Goodness of Fit Index
TLI: Tucker-Lewis Index
CR: Composite Reliability
C.R.: Critical Ratio
AVE: Average Variance Extracted.
