# Supplementary material for: Enacting the curriculum: The interplay of pedagogical beliefs, motivation, and fidelity
Source: PLoS One. 2026 May 13;21(5):e0349102. doi: 10.1371/journal.pone.0349102 (PMC13170868; doi:10.1371/journal.pone.0349102)
Supplement: S1 Table — (DOCX) [file pone.0349102.s001.docx]

|  |  |  | **N** | **X** | **SD** | **F** | **p** |
| --- | --- | --- | --- | --- | --- | --- | --- |
| **Age** | CFS | 20-24 | 15 | 3,722 | 0,466 | 0,307 | 0,873 |
|  |  | 25-29 | 125 | 3,663 | 0,492 |  |  |
|  |  | 30-34 | 105 | 3,680 | 0,481 |  |  |
|  |  | 35-39 | 117 | 3,629 | 0,421 |  |  |
|  |  | 40+ | 62 | 3,624 | 0,512 |  |  |
|  | MTS | 20-24 | 15 | 4,058 | 1,219 | 0,851 | 0,493 |
|  |  | 25-29 | 125 | 4,199 | 0,884 |  |  |
|  |  | 30-34 | 105 | 3,994 | 1,056 |  |  |
|  |  | 35-39 | 117 | 4,099 | 0,952 |  |  |
|  |  | 40+ | 62 | 3,968 | 1,119 |  |  |
|  | TLCS | 20-24 | 15 | 3,511 | 0,512 | 1,159 | 0,328 |
|  |  | 25-29 | 125 | 3,639 | 0,402 |  |  |
|  |  | 30-34 | 105 | 3,574 | 0,349 |  |  |
|  |  | 35-39 | 117 | 3,539 | 0,438 |  |  |
|  |  | 40+ | 62 | 3,534 | 0,510 |  |  |
| **Experience** | CFS | 0-4 years | 153 | 3,663 | 0,535 | 0,071 | 0,991 |
|  |  | 5-9 years | 114 | 3,644 | 0,424 |  |  |
|  |  | 10-14 years | 74 | 3,661 | 0,510 |  |  |
|  |  | 15-19 years | 51 | 3,631 | 0,382 |  |  |
|  |  | 20+ years | 32 | 3,671 | 0,345 |  |  |
|  | MTS | 0-4 years | 153 | 4,186 | 0,948 | 0,929 | 0,447 |
|  |  | 5-9 years | 114 | 3,950 | 1,051 |  |  |
|  |  | 10-14 years | 74 | 4,071 | 0,828 |  |  |
|  |  | 15-19 years | 51 | 4,091 | 1,106 |  |  |
|  |  | 20+ years | 32 | 4,066 | 1,173 |  |  |
|  | TLCS | 0-4 years | 153 | 3,628 | 0,450 | 1,030 | 0,391 |
|  |  | 5-9 years | 114 | 3,540 | 0,419 |  |  |
|  |  | 10-14 years | 74 | 3,548 | 0,397 |  |  |
|  |  | 15-19 years | 51 | 3,572 | 0,424 |  |  |
|  |  | 20+ years | 32 | 3,516 | 0,337 |  |  |
| **Work context** | CFS | Preschool | 32 | 3,719 | 0,559 | 0,410 | 0,746 |
|  |  | Primary school | 141 | 3,669 | 0,494 |  |  |
|  |  | Middle school | 92 | 3,655 | 0,431 |  |  |
|  |  | High school | 159 | 3,628 | 0,456 |  |  |
|  | MTS | Preschool | 32 | 4,246 | 0,973 | 0,381 | 0,767 |
|  |  | Primary school | 141 | 4,097 | 1,030 |  |  |
|  |  | Middle school | 92 | 4,038 | 1,065 |  |  |
|  |  | High school | 159 | 4,061 | 0,930 |  |  |
|  | TLCS | Preschool | 32 | 3,529 | 0,374 | 1,698 | 0,167 |
|  |  | Primary school | 141 | 3,541 | 0,440 |  |  |
|  |  | Middle school | 92 | 3,541 | 0,420 |  |  |
|  |  | High school | 159 | 3,635 | 0,413 |  |  |
| **Major** | CFS | Preschool teacher | 38 | 3,800 | 0,460 | 0,898 | 0,542 |
|  |  | Primary school teacher | 103 | 3,670 | 0,508 |  |  |
|  |  | Social sciences | 66 | 3,602 | 0,497 |  |  |
|  |  | Foreign language | 27 | 3,671 | 0,441 |  |  |
|  |  | Turkish | 32 | 3,596 | 0,546 |  |  |
|  |  | Mathematics | 36 | 3,590 | 0,443 |  |  |
|  |  | Science | 30 | 3,725 | 0,413 |  |  |
|  |  | Special education | 30 | 3,541 | 0,432 |  |  |
|  |  | Physical education | 11 | 3,818 | 0,509 |  |  |
|  |  | Vocational courses | 32 | 3,664 | 0,408 |  |  |
|  |  | Information technologies | 9 | 3,562 | 0,220 |  |  |
|  |  | Arts | 10 | 3,653 | 0,441 |  |  |
|  | MTS | Preschool teacher | 38 | 4,391 | 0,783 | 1,638 | 0,086 |
|  |  | Primary school teacher | 103 | 4,089 | 1,022 |  |  |
|  |  | Social sciences | 66 | 4,055 | 0,910 |  |  |
|  |  | Foreign language | 27 | 4,130 | 1,120 |  |  |
|  |  | Turkish | 32 | 4,070 | 1,020 |  |  |
|  |  | Mathematics | 36 | 3,882 | 1,104 |  |  |
|  |  | Science | 30 | 3,979 | 0,980 |  |  |
|  |  | Special education | 30 | 3,642 | 1,240 |  |  |
|  |  | Physical education | 11 | 4,580 | 0,725 |  |  |
|  |  | Vocational courses | 32 | 4,363 | 0,574 |  |  |
|  |  | Information technologies | 9 | 4,139 | 0,858 |  |  |
|  |  | Arts | 10 | 3,775 | 1,369 |  |  |
|  | TLCS | Preschool teacher | 38 | 3,564 | 0,353 | 1,365 | 0,187 |
|  |  | Primary school teacher | 103 | 3,553 | 0,464 |  |  |
|  |  | Social sciences | 66 | 3,563 | 0,491 |  |  |
|  |  | Foreign language | 27 | 3,602 | 0,447 |  |  |
|  |  | Turkish | 32 | 3,555 | 0,318 |  |  |
|  |  | Mathematics | 36 | 3,516 | 0,350 |  |  |
|  |  | Science | 30 | 3,628 | 0,314 |  |  |
|  |  | Special education | 30 | 3,414 | 0,342 |  |  |
|  |  | Physical education | 11 | 3,621 | 0,640 |  |  |
|  |  | Vocational courses | 32 | 3,760 | 0,382 |  |  |
|  |  | Information technologies | 9 | 3,787 | 0,433 |  |  |
|  |  | Arts | 10 | 3,633 | 0,399 |  |  |
|  |  |  | **N** | **X** | **SD** | **t** | **p** |
| **Gender** | CFS | Female | 200 | 3,637 | 0,483 | -0,710 | 0,478 |
|  |  | Male | 224 | 3,670 | 0,461 |  |  |
|  | MTS | Female | 200 | 4,134 | 0,987 | 1,013 | 0,312 |
|  |  | Male | 224 | 4,036 | 1,002 |  |  |
|  | TLCS | Female | 200 | 3,587 | 0,385 | 0,525 | 0,600 |
|  |  | Male | 224 | 3,565 | 0,453 |  |  |

**S1 Table.** Results generated from comparative analyses of teachers' curriculum fidelity, teaching motivations, and pedagogical beliefs based on demographic variables.
